# Integrated Approach in Genomic Selection to Accelerate Genetic Gain in Sugarcane

**DOI:** 10.3390/plants11162139

**Published:** 2022-08-17

**Authors:** Karansher Singh Sandhu, Aalok Shiv, Gurleen Kaur, Mintu Ram Meena, Arun Kumar Raja, Krishnapriya Vengavasi, Ashutosh Kumar Mall, Sanjeev Kumar, Praveen Kumar Singh, Jyotsnendra Singh, Govind Hemaprabha, Ashwini Dutt Pathak, Gopalareddy Krishnappa, Sanjeev Kumar

**Affiliations:** 1 Department of Crop and Soil Sciences, Washington State University, Pullman, WA 99163, USA; 2 Division of Crop Improvement, ICAR-Indian Institute of Sugarcane Research, Lucknow 226002, India; 3 Horticultural Sciences Department, University of Florida, Gainesville, FL 32611, USA; 4 Regional Center, ICAR-Sugarcane Breeding Institute, Karnal 132001, India; 5 Division of Crop Production, ICAR-Sugarcane Breeding Institute, Coimbatore 641007, India; 6 Division of Crop Improvement, ICAR-Sugarcane Breeding Institute, Coimbatore 641007, India

**Keywords:** genomic selection, prediction models, GEBV, genomic accuracy, sugarcane, breeding, high-throughput phenotyping, high-throughput genotyping, machine learning, speed breeding

## Abstract

Marker-assisted selection (MAS) has been widely used in the last few decades in plant breeding programs for the mapping and introgression of genes for economically important traits, which has enabled the development of a number of superior cultivars in different crops. In sugarcane, which is the most important source for sugar and bioethanol, marker development work was initiated long ago; however, marker-assisted breeding in sugarcane has been lagging, mainly due to its large complex genome, high levels of polyploidy and heterozygosity, varied number of chromosomes, and use of low/medium-density markers. Genomic selection (GS) is a proven technology in animal breeding and has recently been incorporated in plant breeding programs. GS is a potential tool for the rapid selection of superior genotypes and accelerating breeding cycle. However, its full potential could be realized by an integrated approach combining high-throughput phenotyping, genotyping, machine learning, and speed breeding with genomic selection. For better understanding of GS integration, we comprehensively discuss the concept of genetic gain through the breeder’s equation, GS methodology, prediction models, current status of GS in sugarcane, challenges of prediction accuracy, challenges of GS in sugarcane, integrated GS, high-throughput phenotyping (HTP), high-throughput genotyping (HTG), machine learning, and speed breeding followed by its prospective applications in sugarcane improvement.

## 1. Introduction

Recent advancements in the field of plant breeding and crop-raising practices have profoundly contributed to the tune of 0.8–1.2% annual crop yield rate increase for major crop plants. However, the current rate of productivity gain is not sufficient, and ~2.4% yield gain is required to meet the expected global food demand by 2050 [1]. Relying only on conventional breeding may not be sufficient to realize the required rate of genetic progress, particularly, in the era of climate change and continuously depleting land and water resources. The problem is further complicated by the genetic nature of yield and its component traits, as these are greatly affected by environmental factors, which make their improvement slower and more protracted by conventional breeding approaches. The genetic improvement of crops was primarily through conventional breeding methodologies until the 1980s, and such methods are still a common choice for plant breeders; however, genetic progress is limited, especially for complex and environmentally sensitive traits such as yield (and its component traits) and tolerance to abiotic stresses [2].

Approximately 80% of global sugar production comes from sugarcane crop, and millions of farm workers, in addition to sugar factory/mill workers, directly or indirectly depend upon sugarcane agriculture. The modern cultivars of sugarcane (*Saccharum* species hybrid) are the outcome of inter-specific hybridization between domesticated octaploid *S. officinarum* (2n = 80; x = 10) and wild *S. spontaneum* (2n = 40–128; x = 8) with minor contributions from *S. robustum*, *S. sinense*, and *S. barberi* and related genes such as *Miscanthus*, *Narenga*, and *Erianthus*. Due to this, the cultivated varieties have a highly complex polyploid genome and exhibit a high level of heterozygosity coupled with varied number (100–130) of chromosomes. Sugarcane has one of the most challenging genomes of all crops; it is an auto- and allo-polyploid species with a high level of aneuploidy [3]. With chromosomal numbers varying among accessions from 80 to >100, dozens of copies for each homoeologous chromosome, and an estimated total genetic map length of 17,000 cM, it represents a formidable challenge to any genotyping technology. However, one of the advantages with sugarcane is that vegetative propagation facilitates fixation of traits in successive clonal generations. Recent genome-wide association studies (GWAS) revealed that most common genetic variants could explain only a small proportion of the variance of a complex trait [4,5]. The overall sugarcane productivity is affected by numerous factors; the most important of which are sugar content, yield, and resistance/tolerance to biotic (especially red rot disease) and abiotic stresses (such as drought and water logging). Sucrose is the prime product of sugarcane, and a significant variation has been reported among different sugarcane genotypes. High sucrose accumulation in sugarcane at an early crop phase is one of the most desirable traits, since it can help in reducing its long growth cycle. This is the main reason that genetic improvement efforts in sugarcane are largely centered around increasing its sucrose accumulation potential. Finding markers/quantitative trait loci (QTLs) that are closely associated with sugar- and yield-associated traits would enhance the precision and time taken for clone selection in sugarcane, which is exclusively a vegetative propagated long-duration crop. The two main approaches, map-based and association-based, are in practice meant to find markers associated with traits of interest [5]. The highly heterozygous and polyploid sugarcane genome, however, is a bottleneck in analyzing trait inheritance.

Sugarcane breeding programs, at present, heavily rely on phenotypic selection, which is usually based on combined mass- and family-selection strategies. Improvements in yield- and disease resistance- associated traits require large experiments for many crop cycles [6]. It takes 8–10 years of field evaluations to identify elite clones for further multi-location trials before they can be released for commercial cultivation [7,8]. Since the early 1990s, marker-assisted selection (MAS) has been used in a number of commercial crop breeding programs for the tagging of genes associated with economically important traits, which has enabled the development of a number of superior cultivars in various crops. Marker-assisted selection has been applied successfully in a number of crops [9,10], but genome complexities have limited the application of MAS in sugarcane. The use of molecular markers in sugarcane breeding has only been used to tag a few major genes associated with disease resistance [11,12,13].

The majority of QTL studies have common features, such as the modest phenotypic effect of QTLs and a lack of repeatability across environments or crop cycles. Against such a backdrop, validating common QTLs between studies is not easy for agronomic and yield-associated traits. Nevertheless, MAS [9], marker-assisted backcrossing (MABC), and marker-assisted recurrent selection (MARS) [14] continue to be used to fix the shortcomings linked with commercial varieties through gene pyramiding and to enrich popular varieties with new alleles. However, the incorporation of a few alleles/genes is not sufficient to improve the complex/quantitative traits, which are governed by a number of minor effect QTLs [15]. Furthermore, the efficiency of MAS in the genetic enhancement of quantitative traits is limited due to the use of low/medium-density markers. The main gap in the applicability of MAS in sugarcane is due to the low percentage of trait variation explained by each individual marker. This could be attributed to the high ploidy level of the sugarcane genome, wherein each trait is governed by the allelic dosage at the trait locus/loci and only the extreme genotypes are tagged in the whole allelic series. Hoarau and co-workers, in a QTL study, opined that the modern sugarcane cultivars have accumulated more favorable alleles that have resulted in diminishing the internal contrast that determines trait segregation and the magnitude of QTL effects [16]. MAS-based breeding is limited by several factors, for example, marker analyses generally miss small-effect loci, and thus, only a few large-effect loci are considered that may not capture all genetic variation for the trait [17,18]. In addition, such programs employ a relatively small number of individuals (250–300) in mapping populations to identify QTLs, which leads to inflated QTL effects [19,20]. Considering the above peculiarities of sugarcane, genomic selection (GS) could be the most promising breeding approach for improving complex traits.

The main strength of GS is that it can capture several small-effect genetic factors and can also improve multiple traits simultaneously. The selection of genotypes in GS is based on genomic estimated breeding values (GEBVs), which have great potential in enhancing selection efficiency. GS has a clear edge over pedigree selection and MAS, particularly in the improvement of complex traits such as yield in a short span of time by the reduction in number of breeding cycles [21]. Speed breeding (SB) in crops is becoming an effective breeding strategy to shorten the breeding cycle, resulting in rapid generation advancement [22,23]. An integration of GS and speed breeding could accelerate the mining of untapped novel genes/alleles for rapid genetic progress in crops [24]. For better understanding of the GS integration, the concept of genetic gain is comprehensively discussed through the breeder’s equation, GS methodology, prediction models, status of GS in sugarcane and challenges in its application, prediction accuracy, high-throughput phenotyping, high-throughput genotyping, machine learning, and speed breeding. In addition, the prospect of GS in sugarcane improvement is also reviewed. Some of the earlier reviews have also highlighted the importance of GS for crop breeding [25,26,27], especially Varshney and associates emphasizing the role of the five Gs (genome assembly, germplasm characterization, gene function identification, genomic breeding, and gene editing) for crop genetic improvement [25]. The five Gs covered MAS, MABC, and MARS for crop improvement. In addition to these genomic breeding methodologies, novel approaches (such as forward breeding, haplotype-based breeding, and genomic selection) coupled with speed breeding are also discussed. The same group, in another review, highlighted the role of genomics-assisted breeding in designing future crops [26]. The crop wild relatives (CWRs) are very important for genetic studies and breeding; new breeding tools such as GS and optimum contribution selection may help to achieve the best combinations of beneficial alleles in exotic × elite crosses, thereby, enhancing the genetic potential. Thorough and systematic analysis of gene bank collections could guide future germplasm collection strategies [27]. Similarly, the much less explored area of sugarcane cystatins was also highlighted [28], as cystatin engineering will be helpful to increase yield and stability and improve their tridimensional conformation, which may open new opportunities for their application in sugarcane improvement. Furthermore, interactions between expression of the gene(s) and physiological changes during water stress have also been reviewed, wherein, the role of ABA signaling pathways, proline (as an important drought stress osmoprotectant), and aquaporin proteins (as a potential source of gene manipulation in sugarcane) were emphasized [29]. Although, these reviews cover GS and related aspects [25,26,27,28,29], most of them are either too general, covering multiple crops and topics, or too specific. Further, machine learning has not been covered in these reviews. The present review is unique as it highlights integration of phenomics, genomic selection, and machine learning for sugarcane improvement. The sugarcane genome is much more complex compared to diploid and other polyploid crops, and less amenable to genomic-assisted breeding (GAB). Therefore, a thorough discussion of machine learning along with GS and phonemics could be rewarding.

## 2. The Breeder’s Equation and Genetic Gain

Genetic gain in a crop breeding program is determined by the following popular breeder’s equation:

∆G=ihσa/L

where, *i* is the selection intensity, *h* is the narrow sense heritability, *σa* is the additive genetic variance, and L is the length of the breeding cycle interval or generation.

Genetic gain per unit time can be accelerated by reducing the length of the breeding cycle interval. Increasing the breeding cycles per unit time and reducing the cost of phenotyping would greatly increase genetic gain in GS [30]. A higher rate of annual genetic gain could be realized under a reduced breeding cycle, particularly for long-breeding-cycle plant species like sugarcane [31]. Accelerating the breeding cycles in unit time can increase the recurrent selection [32]. GS facilitates the rapid selection of superior genotypes and accelerates the breeding cycle [21,30].

## 3. Genomic Selection Methodology

In 2001, Meuwissen and associates were the first to demonstrate GS as an approach to capture total additive genetic variance using genome-wide molecular markers [33]. Although GS has been practiced extensively in animal breeding for a long time [34], in plants and tree species, the approach is still not well established [35,36]. Unlike the marker-based approach, wherein a single marker/QTL is focused, GS relies on the prediction of individual phenotypic expression, in terms of breeding value or genetic worth of an individual based on genome-wide marker data through GEBV estimation. This is achieved by using a prediction model trained with a representative set of individuals whose genotyping and phenotyping have been carried out; this group of individuals are referred to as the ‘training population’ (TP). Unlike traditional marker-assisted breeding, GS does not depend on a group of polymorphic and linked markers; the calibrated/trained models in GS have the potential to capture a greater portion of genotypic variation by considering the minor QTL effects [33]. One of the advantages of GS is that the individuals whose GEBVs have been estimated can be phenotyped after selection and crossing, while advancing the superior breeding lines based on GEBVs per se, thereby enhancing the genetic progress per unit time compared to phenotypic selection. In GS, marker effects are estimated individually throughout the genome of the ‘breeding population’ (BP) on the basis of the predictive model trained in the TP. The ‘training population’ is both genotyped and phenotyped, whereas, the BP is only genotyped but not phenotyped; thus, TP is used to train/calibrate the statistical prediction models to predict the breeding values of BP. The ‘breeding population’ should ideally constitute the TP descendants or elite breeding lines that are closely related to TP. Generally, the TP consists of a group of closely related individuals, such as half-siblings with known descendancy. The performance of different traits in the BP are determined by the allelic similarity with loci that are associated with the phenotype in the TP. Thus, GS relies on the level of genetic resemblance between TP and BP in the linkage disequilibrium (LD) between marker and trait loci [37]. Diverse, extensively phenotyped and genotyped lines from a breeding program would be a potential TP for robust calibration of prediction models [38]. The GEBV is the estimation of the breeding value of BP (genotyped) using TP (genotyped and phenotyped) through statistical modeling. GEBV is derived by the combination of desirable loci across the individual genome of the BP. Superior lines selected based on high GEBVs in the BP would further serve as potential parents in a breeding program, without testing their phenotypic performance in the field conditions.

## 4. GS Prediction Models

Although, many GS models have been designed to predict genotypic performance in crops, the selection of a suitable statistical model is essential to obtain a relatively higher predictive ability and thereby the success of GS. The assumptions and treatments of marker effects decide the level of prediction accuracy in different GS models [39,40]. The two most commonly used predictive models in different GS studies in crops are genomic best linear unbiased prediction (G-BLUP) and ridge regression best linear unbiased prediction (RR-BLUP). The RR-BLUP assumes that all markers have equal variances with small but non-zero effect; however, this assumption of equal variance does not imply that the effects of all markers are equal [35]. If the trait is controlled by a large number of loci with each locus having small effect, then the RR-BLUP model results in relatively higher predictive abilities [41]. Another extensively studied model is G-BLUP, which uses genome-wide markers to predict the genetic and phenotypic values of selection candidates [42]. Both the G-BLUP and RR-BLUP models have common assumption that the effects of all loci have a common variance, which makes them more suitable for traits influenced by a large number of minor genes. However, the G-BLUP and RR-BLUP assumptions are seldom met as most of the markers in the whole genome have small or no effects and a few markers have large effects. Most of the Bayesian models (Bayes A, Bayes B, Bayes Cπ, LASSO) fit into the real conditions and allow different markers to have different effects and variances. Bayes A is mostly suitable for traits governed by a moderate number of genes as the shrinkage level is weaker compared to Bayes B and Bayes Cπ. Bayes B model assumes that most loci have no effect on the trait, and thus most markers are left out of the prediction model. Bayes B fits well if the trait expression is governed by large-effect QTLs that explain much of the genetic variation [43]. In contrast, in Bayes Cπ, the parameter π can be calculated on the basis of experimental data, and thus the shrinkage level is estimated. Therefore, it is more suitable than Bayes B for real data analysis. Bayesian models will generally have better prediction accuracies as they capture large-effect QTLs. Bayesian LASSO integrates the features of subset selection with the shrinkage produced by Bayesian regression. Reproducing Kernel Hilbert Space (RKHS) integrates an additive genetic model with a kernel function and converts predictor variables to a set of distances among observations to produce a definite matrix to be used in a linear model [44]. Selective shrinkage models, such as Bayes B, Bayes Cπ, Bayes A, and Bayesian LASSO, are sensitive to the number of QTLs: the predictive ability decreases with increase in the number of QTLs [42]. On the other hand, the predictive ability of G-BLUP and RR-BLUP often stays nearly constant regardless of the number of QTLs, so it is more feasible for plant traits governed by a larger number of minor genes.

## 5. Current Status of GS in Sugarcane

Gouy and associates opined that in a highly polyploid crop like sugarcane, larger panel and high-throughput genotyping may bring out interesting new revelations if GS is applied to the populations that are otherwise rejected in the first stage [4]. In addition, recent advancements in the genomics in polyploid systems have provided more efficient markers, such as SNPs, which can cover the whole genome [45,46]. These marker systems have the capacity to utilize the allelic dosage data with a better statistical algorithm, which can account for the ambiguities of the sugarcane genome more efficiently. Gouy and associates reported genomic selection in sugarcane for the first time [4]. A panel of 167 sugarcane clones was screened with 1499 diversity array technology (DArT) markers and phenotyped for 10 traits (sugar content, bagasse traits, morphological traits), including 3 diseases (smut, brown rust, and yellow leaf virus) [4]. In this study, small to moderate levels of accuracy (0.11–0.62) with large variation between different traits was observed (Table 1). However, the study included a relatively small number of markers and a small training population size. Nevertheless, in view of the accuracy of prediction for some of the traits, the study was encouraging in terms of potential for practical application in sugarcane breeding.

Breeding processes in sugarcane are challenging due to the factors discussed above, therefore, sugarcane breeding is heavily dependent on phenotypic selection. This necessitates large-scale field trials and several cycles of selections spanning the whole process over 8–10 years. Olatoye and co-workers studied the abilities of GS and MAS to predict traits under different genetic architectures and marker densities [47]. Due to the lack of genomic data for *Miscanthus* × sugarcane hybrids, *Miscanthus* × *Miscanthus* and sugarcane × sugarcane F_1_s and BC_1_ populations were analyzed. GS yielded a higher prediction accuracy, identified more genotypes with the best-performing simulated trait values, and more accurately predicted the traits. Although, none of the GS models decisively outperformed the others, MAS was suggested to be a reasonable option for advancing vertical disease resistance. Deomano and co-workers assessed genomic prediction accuracy for cane yield and sugar content using three different commercial sugarcane populations consisting of 467, 1146, and 738 clones in different stages of selection trials [48]. It was concluded that the prediction models coupled with marker data had higher prediction accuracies compared to that of the models using only the pedigree data [48]. Aono and co-workers employed eight different machine learning models to establish a subset of SNPs with good ability to predict brown-rust-resistant phenotypes (Table 1) [49]. Similarly, Hayes and associates used an extra-large (3984 individuals) population [50]. Islam and associates generated genotypic data from 432 sugarcane clones using target enrichment sequencing, and a set of 8825 SNPs markers to assess the prediction accuracy of multiple GS models for brown and orange rust resistance [51]. The models included random regression BLUP (RR-BLUP) with and without known locus, reproducing kernel Hilbert space (RKHS), random forest (RF), and support vector regression (SVR) (Table 1). The GS prediction accuracies for brown rust and orange rust were in the ranges of 0.28–0.43 and 0.13–0.29, respectively. Furthermore, the inclusion of a known major gene for resistance to brown rust as a fixed effect in the GS model substantially reduced the minimum number of markers and training population size. Vos-Fels and associates tested two GS schemes, rapid recurrent genomic selection with or without phenotyping, and reported that both simulated GS schemes achieved genetic gains of 2.6–2.7%, which were ~2x higher compared to the phenotypic selection scheme (1.4%) [52].

## 6. Prediction Accuracy of the GS Model

Most of the genetic gains of GS come from obtaining accurate predictions in early stages of the breeding cycle and shorter breeding cycles. The genomic prediction accuracy is affected by several factors: genetic relatedness [53], marker type and density [37,54], trait heritability [55,56], effects of genes (additive/non-additive gene action/gene interactions) [57,58,59], size of the population [60], structure of the population [61], predictive models used to calibrate the best-fitted model [62], extent and distribution of LD, and genotype–environment interaction [63]. The prediction accuracy of the standard GS model has been reported to have a positive association with heritability [64]. The composition of the TP in relation to the BP is important to obtain a high degree of GS accuracy [37]. Pooling of related populations increases the prediction accuracy in GS models [53]. Population structure will have an effect on the genomic-wide predictions [65]. Gouy and associates reported that a high level of LD (5 cM) and the type of markers used are the major factors that affect accuracy of GS prediction [4].

### Challenges in Applying GS in Sugarcane

Compared to other crops, sugarcane undergoes various growth phases—germination (0–60 days), formative phase (60–150 days), grand growth phase (150–240 days), and maturity phase (240–360 days)—thus facing weather variations for a longer period. Hence, phenotyping of a large number of genotypes becomes a challenge, especially considering different growth phases. Sugarcane possess one of the most challenging genomes of all crops (estimated genetic map length: 17,000 cM) posing a formidable challenge to genotyping techniques. In addition, the population and unique genetic parameters also pose challenges to the adoption of GS models, which have been primarily developed for seed-propagated crops. For example, a majority of the GS models consider the additive effects and assume dominance and epistatic effects as residual. For clonally propagated crops like sugarcane, however, dominance and epistatic effects play an important role in addition to additive effects. This hold true as the whole set of alleles, together with their interactions, are passed to the next generation through clonal propagation. Another possible source of variation that is not accounted for could be additive effects due to varying allele dosages. Since SNPs are only defined as being present or absent, in sugarcane, variation in the numbers of copies of each allele may be important. In addition, data from different stages of the selection may have implications on the accuracy of genomic prediction.

## 7. Integrated GS: Novel Tools Supplementing GS

**Figure 1:**
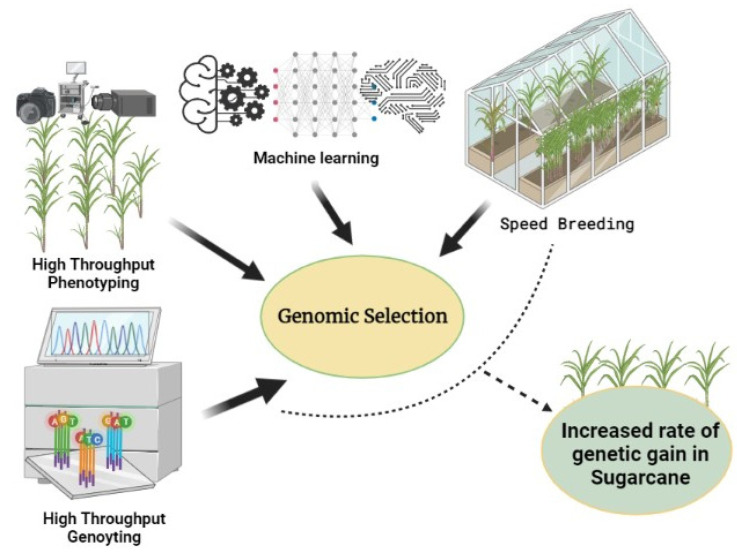
Various approaches which could be integrated in genomic selection in sugarcane for accelerated genetic gain.

Since a large population needs to be phenotyped as well as genotyped, GS may need to be integrated with high-throughput technologies and statistical robustness. This necessitates a thorough understanding of sugarcane phenotype at various growth phases so as to integrate the complex pathways and the physiological and biochemical traits. In recent times, hyperspectral cameras and mechanical devices have been developed by which one can study complex traits much faster and in a more precise manner. The long-read sequence platforms such as the PacBio Single-Molecule Real-Time (SMRT) system, Illumina TruSeq, and Oxford Nanopore sequencing could solve the problem of genome assembly. Furthermore, integration of NGS techniques with machine learning and deep learning tools would not only facilitate robust SNP mining but also GS model development and their validation in a high-throughput fashion to increase the prediction accuracy. Finally, speed breeding (SB), once standardized for sugarcane, could be effectively utilized to increase the rate of genetic gain by reducing the length of the selection cycle. In a complex crop like sugarcane, the full potential of GS could be realized by an integrated approach combining high-throughput phenotyping, genotyping, machine learning, and speed breeding (Figure 1).

### 7.1. High-Throughput Phenotyping in GS

**Figure 2:**
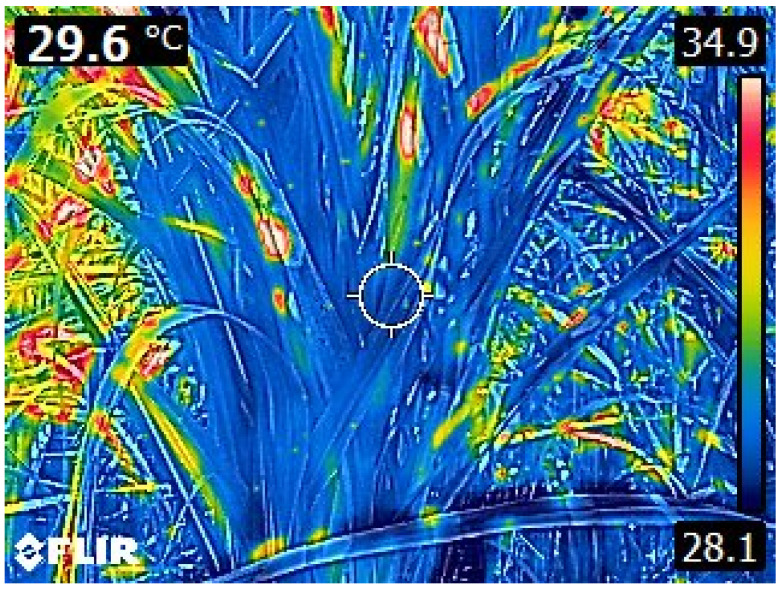
Infrared thermal image of canopy temperature of a sugarcane plant.

Sugarcane, a C_4_ crop, is considered as a high-biomass crop that is efficient in harvesting sunlight and converting the carbon dioxide to sucrose through complex redox reactions; it also produces various complexes of carbohydrates, lignin and fiber. The looming dangers of climate change coupled with increasing population and food insecurity have led the focus toward sustainable crop production that does not affect the environment. Compared to other crops, sugarcane is a complex one that undergoes various phenophases, as mentioned above [66], and it faces extreme weather variations for a longer period. Hence, a thorough understanding of sugarcane phenotype at various growth phases would be very useful for their improvement. With the advent of new spectral cameras and automatic mechanical devices such as drones during the last decade, researchers are now able to study complex traits much faster, and more precisely and accurately [67]. These advancements are playing a major role in the recording of valuable crop growth and development of sugarcane, which are tedious when using routine manual methods. Recently, Yang and co-workers emphasized that advanced plant phenomics would enable effective use of germplasm, novel gene discovery, and improved crop yield and quality [68]. Various spectrums of light—red blue green (RGB), infrared rays (IR), near-infrared rays (NIR), and hyperspectral images—are becoming useful tools for rapid phenotyping of a large number of sugarcane genotypes. For example, Figure 2 depicts an infrared thermal image of the canopy temperature of a sugarcane plant, wherein, the temperature difference is clearly seen.

#### 7.1.1. Cane Yield

In sugarcane, the cane yield is observed as a function of the stalk population per unit area (number of millable canes; NMCs) and single cane weight. The foremost component of yield is the number of tillers at harvest, and having a higher correlation coefficient, it is directly associated with yield [69]; cane length also had positive associations with yield (0.48 and 0.53, respectively) [70]. Rosario and associates studied the association of sugar yield and its components to physiological (net carbon exchange; NCE), chlorophyll “a”, “b”, and total chlorophyll content; protein content; phosphorous and potassium content in leaf; and morphological traits (leaf angle, leaf width, specific leaf weight) in 14 sugarcane varieties and reported that the net carbon exchange had a significant positive correlation with cane length, number of tillers, and NMCs [71]. They also reported that both fresh and dry weight possess good correlations with NCE, which in turn results in better competitive ability with erect and narrow leaves for population stress [71]. Sugarcane varieties with narrow, erect, and thick leaves exhibit early and rapid vegetative growth, with more light interception and leaf production along with better growth rates. They also reported that the selection of better stalk diameter canes for more yield often results in lower NMCs [71]. In sugarcane, the yield is a polygenic trait, and there are reports confirming the significant negative association between yield and quality, which are the two prime considerations for sustaining sugarcane productivity under a changing climate scenario.

##### Determination of Cane Yield through Phenomics Approach

Sugarcane is a multifunctional crop primarily used for sugar and renewable bioenergy production, and precise and timely assessment of the sugarcane yield before harvest plays a predominant role in the supervision of agroecosystems [72]. LiDAR mounted on an unmanned aerial vehicle (UAV) combined with random forest regression (RFR) prediction model resulted in higher prediction accuracy in estimating the sugarcane above-ground fresh weight, with the observed value of R^2^ = 0.97. A successful (>90% accuracy) forecasting model for pre-harvest sugarcane yield determination using UAV-acquired RGB color images coupled with ground information data has also been reported [73]. Recently, a new robust sugar cane model and classification method were reported, which correctly forecasted total sugarcane harvest yield with a superior accuracy of 98.69% [74].

#### 7.1.2. Stalk Quality

Based on systematic near-infrared spectroscopy, it has been reported that stalk quality in terms of soluble sugar, insoluble residues, and the corresponding fundamental ratios can be assessed in high-throughput mode [75]. Further, it was stressed that NIR-based high-throughput phenomics can be used for large-scale screening of optimal sugarcane germplasm for stalk quality, etc. Relative water content (RWC), chlorophyll content, canopy temperature depression (CTD), stomatal conductance, early-stage traits, and root phenotyping are considered a few of the important physiological traits that have significant correlations with cane yield and sugarcane productivity. The importance of these aforementioned parameters is highlighted and discussed in the light of phenomics.

#### 7.1.3. Relative Water Content, Water Potential, Water Content

Considering cellular water deficits and their physiological significance, relative water content (RWC) is perhaps the most useful indicator of plant water status. The RWC protocol by Barrs and Weatherly in 1962 still has the highest relevance for studying the plant water status for drought research in crop plants, as shown in Equation (1) [76].

RWC = [(Fresh weight − dry weight)/(Turgid weight − dry weight)] × 100(1)

In sugarcane, the RWC of transpiring leaves ranges from 60 to 85%. A lower value of RWC is observed in plants grown under water-limited conditions and vice versa under irrigated conditions. The RWC and water potential plays a major role in sugarcane under drought stress signifying cellular water status, establishing cellular processes, and eventually, sugarcane productivity. Nowadays, through a plant phenomics facility, the RWC/water content/water potential can be derived easily with an NIR camera by creating indices that could facilitate the screening of a large number of genotypes for drought tolerance and sugarcane productivity. Ripple emphasized the significant correlation between leaf water potential and reflectance [77]. The vibration processes of O–H bonds of the water molecule at 975, 1175, 1450, and 1950 nm play a major role in plant reflectance spectroscopy [78]. Hyperspectral sensors are increasingly becoming more useful in assessing plant water status through reflectance measurements [79,80,81].

#### 7.1.4. Canopy Temperature

In well-watered plants, transpiration helps the plants to adapt to stress conditions. Canopy temperature is generally measured with IR thermometers or thermal gun. Generally, the plant canopy emits long-wave infrared radiation as a function of temperature, and the IR thermal gun records the IR radiation, converts the recorded radiation as an electric signal, and displays the signal as a temperature [82]. The canopy temperature (CT) is commonly used to indicate vegetative water status. With an IR temperature gun, canopy temperature can be measured to determine the degree of stomatal opening; when stomata remain open, plants will be moderately cooler. Canopy temperature or canopy temperature depression (CTD) has been used in many crop-screening programs for various abiotic stresses. Several workers have reported the usefulness of CTD under high-temperature and drought stress conditions [82,83,84]. By increasing evaporation through stomata, leaves lose their effective transpiration efficiency (carbon assimilation per unit of transpiration). In 1990, Singh reported that CTD is an inherited trait that can be used in breeding programs for stress conditions [85]. It has been recently emphasized that CTD in sugarcane clones grown under water-limited conditions had a significant positive correlation with cane yield [86]. In spite of reduced irrigation, a few clones had cooler canopies and positive CTD. These clones may have better root systems for extracting water from deeper in the soil for transpirational cooling of the canopy, resulting in better physiological processes. In general, due to partial or complete closure of the stomata at peak stress, the canopy temperature rises more (negative CTD) in susceptible plants compared to the drought-tolerant plants [67]. Thermal imaging is a promising tool for recording surface temperatures at both the canopy and leaf level, with a better correlation with stomatal conductance in maize [87,88], and also for stress responses from plant pathogens or salinity [89]. Canopy temperature measurements are lengthy and labor-intensive and are heavily influenced by the variation in vapor pressure deficit (VPD) and radiation [90], and they are relatively low throughput, and therefore, unsuitable for screening large populations. Recently, thermal IR cameras were employed for canopy temperature observations in many crops including sugarcane (Figure 2). The UAV-assisted measurements of CT and various agronomic traits were successful in many crops [91,92,93]. Basnayake and associates clearly demonstrated the usefulness of UAV-assisted CT measurements in sugarcane compared to that of traditional lengthy and labor-intensive measurements through hand-held IR camera [94].

#### 7.1.5. Early-Stage Traits in Sugarcane

Based on the field experiment, Natarajan and co-workers suggested the use of various sensors (visual, multispectral, and thermal cameras) mounted on a UAV in high-throughput phenotyping, especially for indirect traits (e.g., canopy cover, height and temperature, and normalized difference vegetation index (NDVI; Equation (2)) [95]. In early-stage sugarcane breeding programs, UAV-assisted phenotyping is viewed as a valuable strategy for improving clonal selections and genetic gains. It was reported that the number of tillers and plant height at six months after planting are highly correlated with canopy cover (rg = 0.72) and canopy height (rg = 0.69), respectively [95].

NDVI = (NIR − Red)/(NIR + Red)(2)

#### 7.1.6. Chlorophyll Content

**Figure 3:**
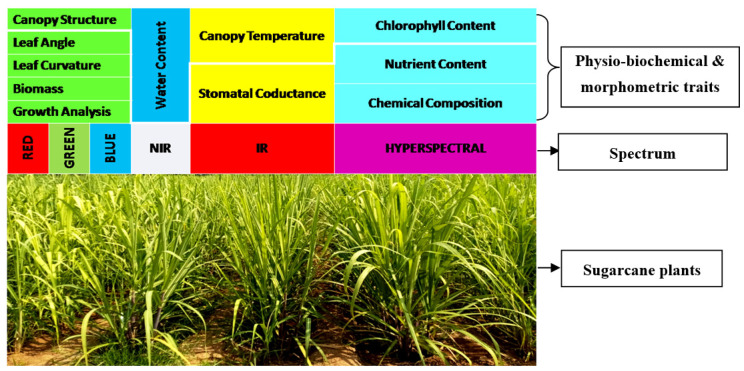
Various spectra under which different physio-biochemical and morphometric traits are measured in sugarcane high-throughput phenomics.

The chlorophyll content is reported to have a significant positive correlation with cane yield. Non-destructive measurement of chlorophyll content through SPAD meter (portable optical meter for measuring the absorbance of red light at 650 nm and infrared light at 940 nm) offers a simple way to determine the chlorophyll content in large populations [96]. Due to scientific advancements, hyperspectral sensors are now available for measuring the chlorophyll content, nutrients, and chemical composition in a rapid and accurate way (Figure 3).

#### 7.1.7. Root Phenotyping in Sugarcane

Roots are the first point of contact of plants with the environment, beginning from the germination of seed/propagules, wherein roots provide anchorage in addition to facilitating the uptake of water and nutrients most essential for plant growth and development. Sugarcane root systems are more complex due to their varied forms and distribution, highly branched superficial roots, positively geotropic buttress roots, and deeply penetrating vertical rope roots, each with distinct functionalities. Roots in sugarcane are unique, as they survive even after harvest of the crop, facilitating ratoon establishment. For sugarcane, being a 12–18 month crop from planting to harvest, root growth is massive for different clones. Plasticity in root morphology, distribution, and physiological aspects are important in determining the crop growth under biotic and abiotic stress situations. The hydraulic properties of sugarcane roots are correlated to stomatal conductance, thereby influencing photosynthetic rate and biomass assimilation [97]. Long-term breeding for high yield and quality might have compromised the resource use efficiency of some sugarcane varieties, which is mostly determined by root system traits. The present impetus lies in identifying unique root phenes and understanding their ‘fitness landscape’, that is, how the crop performance is affected by the external environment vis-a-vis alterations to basic phenotype [98]. The wealth of variability inherent in sugarcane germplasm needs to be explored for identification of useful and robust root phenes that are highly plastic in order to adapt to adverse environmental situations. Facilities and platforms for root phenotyping are still in their infancy in most agricultural crops, and more so in the case of sugarcane.

Operational difficulties in phenotyping sugarcane roots include the sheer size of the root system per se, in addition to the huge cost of establishing advanced facilities for high-throughput underground imaging and data analysis. Phenotyping platforms such as GrowScreen-Rhizo, Phytomorph, GrowScreen-PaGe, RADIX, and RhizoTubes have been demonstrated to be effective for root investigation in several other crops. Imaging techniques including X-ray computed tomography (X-ray CT), magnetic resonance imaging (MRI), positron emission tomography (PET), electrical resistance tomography (ERT), electromagnetic inductance (EMI), and ground-penetrating radar (GPR) aid in non-destructive imaging of roots with minimum damage to the plants [99]. High throughput need not necessarily be expensive, as in a setup for rapid, non-destructive, two-dimensional analysis of the root angle in sorghum, without any sophisticated instruments, ensuring minimal disturbance to plant roots [100]. Shovelomics, or root crown phenotyping, which was initially developed for maize, is one of the most widely used field methods in other crops as well [101]. Researchers in Australia have been successful in developing methods to discriminate between sugarcane root systems and capture its structural diversity [102]. Digital image analysis led to the identification of key traits, including root opening angle, root system total length, average diameter, proportion of roots in each size class, nodal root number, specific root length, and root branching density. Studies at ICAR-Sugarcane Breeding Institute, Coimbatore, India, employ multi-pronged approaches for sugarcane root phenotyping with more focus on in situ sampling such as excavation of roots by trench sampling [103], use of root core sampler [104], raised platforms for root sampling, and in-depth studies under controlled conditions using hydroponic culture facilities [105]. With the advancements in studies on root biology of other cereal crops such as rice, maize, and sorghum, there is ample scope to create high-throughput platforms for sugarcane root studies.

### 7.2. Role of High-Throughput Genotyping in GS

High-throughput genotyping is one of the most crucial components of genomic selection, where the type of marker system, method of genotyping, and genotyping platform does not only affect the prediction accuracy, but also the speed, accuracy, and cost of genotyping. The most suitable marker system considered for high-throughput genotyping is single-nucleotide polymorphisms (SNPs), mainly due to its abundance, genomic coverage, ease in identification, high reproducibility, etc. [106]. The advent of next-generation sequencing (NGS) techniques has brought about a revolution in genotyping by not only increasing the throughput but also reducing the overall cost, making it possible to genotype a large number of individuals in a breeding population. In order to use the high-throughput genotyping technique in sugarcane for GS, Gouy and co-workers in 2013 first implemented diversity array technology (DArT) [4]. Later, in the year 2019, Olatoye and associates used the publicly available SNPs obtained from RNAseq and restriction-site-associated DNA sequencing (RAD-seq) [47]. Further, Deomano and associates used an Affymetrix Axiom SNP array [107], covering 47,803 genome-wide SNPs [48]. Similarly, other research groups also used the Affymetrix Axiom SNP array for genotyping [50,108]. Considering the major drawback of array-based SNP genotyping (i.e., ascertainment bias [109]), Islam and co-workers used an NGS-based target enrichment genotyping technique [51], which has an advantage over SNP array as it has less intrinsic bias owing to non-random sampling of polymorphisms in the population of interest [110]. Recently, GBS has been utilized successfully for genotyping the biparental population of sugarcane [111].

However, GBS in sugarcane with reference to GS is still in its infancy. Still, it is the method of choice for most of the researchers working in different crops [112]. It is a comparatively low-cost method [113], and has the capacity to genotype large segregating as well as non-segregating populations. In recent times, it has become a rapid and accurate method of genotyping [114], and it has also been applied to the crops where the genome is not yet sequenced [115]. Further, Bassi and associates compared various high-throughput genotyping techniques and reported GBS as the most economical one, owing to its low cost (USD 12 per sample) [116]. The major problem faced by most of the researchers in GBS is the missing data [117]; therefore, genotype imputation, which not only deals with missing genotype but also reduces the genotyping cost, needs to be further integrated with GBS [118,119]. In 2017, Technow and Gerke introduced a cost-effective parent-progeny imputation method (based on the pooling of individuals and eliminating the need for extra sequencing coverage), and observed a close match between GEBVs derived from imputed markers and true markers, which indicates the reliability of this method [120]. Since the problem of ascertainment bias associated with SNP array has been resolved to a great extent by utilizing the wild relatives in breeding programs [121], the development of ‘second-generation chip’ would further reduce it to the minimum possible level to broaden its applicability [122]. The combined use of targeted GBS and array-based genotyping has been suggested to increase the prediction accuracy of GS [123]. It is expected that an integration of tools that takes into account allele dosage, second-generation chip, and genotype imputation could be the best strategy for genotyping, not only in highly polyploid crops like sugarcane but in other crop systems as well.

### 7.3. Machine Learning Strategies

The breeding cycles for sugarcane to select superior genotypes can take several years, and genomic selection (GS) is an alternative to reduce time period. A major use of GS is to predict the GEBVs using statistical methods built with markers based on training and a testing population to select a set of promising individuals [50]. However, the prediction accuracy can be affected by many factors such as genome size, ploidy level, QTL, gene interactions, sample size, relatedness, marker density, and model assumptions [124]. In the case of sugarcane, due to genetic complexity, linear regression-based predictive models cannot capture the non-linear characteristics [49]. Moreover, sugarcane has variations in allele dosage and multiple copies of the same allele, which creates phenotypic variation [124]. Machine learning (ML) and deep learning (DL) approaches could provide effective alternatives with higher accuracy [125]. Many statistical methods, including parametric and non-parametric methods, have been developed and utilized to improve the phenotypic predictability of large datasets [126]. Previous studies show that genetic architecture and the heritability of traits impact the accuracy and mean squared error [127], and non-parametric methods perform better when the genetic architecture was due to dominance and epistasis [128,129,130]. Several ML and DL models are often employed with phenomics datasets for predicting traits in GS models in cereals, but limited information from those studies is available for sugarcane. These models have the capability to model complex relationships in the data and usually result in higher prediction accuracies [112,127,131].

Genomic selection was first used in sugarcane breeding with two sets of 167 sugarcane clones with 1499 DArT markers, and the prediction accuracies ranged between 0.11 and 0.62 for ten traits related to plant morphology, digestibility and composition of the bagasse, sugar and bagasse contents, and disease resistance [4]. However, the numbers of markers were not enough to cover 10 Gb of the sugarcane genome. This study tested four methods: two parametric (Bayesian LASSO, ridge regression), one semi-parametric (reproducing kernel Hilbert space; RKHS), and one non-parametric (partial least square regression) [132,133,134,135]. All the methods showed equivalent accuracies for a given trait. Similar results were obtained in other crops where no difference in the model’s performances was observed for predicting different traits, but contrasting results were observed by other authors. GS is being applied in breeding programs with two approaches. The first is early generation selection for rapid generation cycle, which uses additive genetic variance component to predict breeding values for making earlier selections and helps decide new parents without waiting for the complete cycle. The second approach focuses on considering all the variance components, mainly additive, dominance, and epistasis, to predict the total genetic merit of a genotype considering the genetic, environmental, genotype–environment interaction, and weather components in the models [136]. The ML and DL models have shown superior performance to predict the total genetic merit in various crop plants [137,138]. Source codes for the various ML and DL models are presented in Table 2.

Another report showed prediction accuracies from 0.25 to 0.45 for yield and sugar content using three panels with 2351 sugarcane clones and Affymetrix Axiom array-based SNPs [48]. Using pedigree and marker data, five models were tested (BayesA, BayesB, Bayesian LASSO, Bayesian GBLUP, and RKHS) [14,33]. The study showed that predictions with marker information showed better performances than models with only pedigree data. A study by Olatoye and co-workers showed the efficiency of GS for phenotypic traits simulated in F_1_ and BC_1_ populations of *Miscanthus* × *Miscanthus* and sugarcane × sugarcane crosses, showing that GS is preferable to MAS for introgression of genetic sources of horizontal disease resistance from *Miscanthus* to sugarcane. This study used markers comprising 3044 RAD-Seq SNPs and 136 Goldengate SNPs for 85 individuals [47]. Several models were evaluated, including additive, dominance, and epistasis variation, using the sommer R package, BayesA, RKHS, and SVM. To capture additive, dominance, and epistasis variances, sommer fits mixed linear models considering multiple random effects with specific variance-covariance structures [144]. BayesA was implemented using the BGLR R package, RKHS to find a higher prediction accuracy with non-additive variance, and SVMR to minimize the prediction error [135,145].

Recently, Hayes and associates tested the potential of GS using a reference population of 3984 sugarcane clones and 26K SNPs for genotyping [50], reporting prediction values of 0.3 to 0.44 for the cane yield, commercial cane sugar (CCS), and fiber content, with validation predicted across years [50]. This study showed higher GEBV accuracies than those reported by Gouy and co-workers [4] as the reference population was larger [50]. In addition, alternative genomic prediction methods, such as single-step evaluation (GenomicSS) using full pedigree and genomic information, were also evaluated. However, with a large-scale genotyping, a simple GBLUP model will be enough for genetic evaluations, as pedigree information adds only a little accuracy. The accuracy of genomic prediction for the flowering traits was also evaluated, and it was found that it could be helpful for the breeders to choose pairs of clones for crossing that have synchronized flowering. In another study, Islam and associates used 432 sugarcane clones and a set of 8825 SNPs from an NSG-based target enrichment genotyping technique, which has less intrinsic bias due to non-random sampling of polymorphisms in the population, and observed prediction accuracies of 0.28–0.43 and 0.13–0.29 for brown rust and orange rust, respectively [51]. Among the five different models tested, the highest overall prediction accuracies were estimated by machine learning models RF and SVR for brown rust and orange rust, respectively [51]. The support vector regression (SVR) and random forest (RF) methods were used to evaluate machine learning methods for GS as these can capture non-additive variance [129,146]. The SVR function can limit the prediction error by fitting models to reduce residuals [145]. Based on the limited learning for ML and DL models in sugarcane, non-parametric GS methods proved to be better for predicting GEBV and could be used in sugarcane breeding to select progenies in the early stages of the breeding cycle and choose potential parents for crossing based on the predicted breeding values. However, findings from other crop plants can be used to strengthen the fact that ML and DL models performed better at predicting several traits in different plant systems.

### 7.4. Combining Speed Breeding with GS

Sugarcane, being a long-duration crop, requires almost 12–14 years to develop a variety through conventional breeding methods [147]; therefore, efficient and strategic implementation of GS in different stages of the sugarcane breeding program is the need of the hour. This would not only reduce the duration of the breeding cycle and number of selection cycles, but also the number of individuals evaluated in each selection cycle, resulting in an increased rate of genetic gain [148]. Further, speed breeding (SB), which allows accelerated plant development and rapid generation cycling under a controlled environment, has been effectively utilized to increase the rate of genetic gain in crop breeding programs by reducing the length of the selection cycle [23]. The rate of genetic gain using SB could further be accelerated by increasing the selection intensity through high-throughput phenotyping of a large number of genotypes under SB conditions [149]. However, these tools were able to independently contribute to accelerated genetic gain. Several researchers have combined GS and SB, which resulted in further increase in the rate of genetic gain in a number of crops. For example, Jighly and co-workers demonstrated the potential to increase the rate of genetic gain in allogamous crops by combining SB and GS (SpeedGS) [150]. Watson and associates proposed an efficient spring wheat breeding strategy by combining SB with multivariate GS, which was able to accelerate the rate of genetic gain in cereals [151]. Recently, Krishnappa and associates reviewed the possibility of combining GS with SB to accelerate the rate of genetic gain in crop plants [117]. Several researchers have advocated the integration of GS and SB to increase the rate of genetic gain in several crops [152,153,154,155]. Although GS has been implemented in sugarcane by various researchers (Table 1), there is no report of using SB in sugarcane. Therefore, limitations of using SB in sugarcane need to be critically examined in light of the challenges of the crop vis-a-vis the potential of SB. Once sugarcane can be grown under SB conditions, a method of integrating GS and SB must be sought to further accelerate the rate of genetic gain.

## 8. Conclusions

Increasing the rate of genetic gain is essential to meet the ever-increasing global food demand. To achieve rapid genetic gain, advanced breeding tools are required. GS is one such proven technology in animal breeding, and it is also incorporated in plant breeding programs, especially in the ever-expanding private sector. GS could be a promising strategy to accelerate genetic gain per unit time and cost, mostly for traits governed by genes with small and cumulative effects. The optimal integration of GS in active breeding programs faces several challenges. Nevertheless, GS has a clear-cut advantage over the other breeding techniques to enhance genetic gains for complex traits. The implementation of GS for low-heritability traits faces challenges due to high environmental effects, genotype–environment interaction, dominant, and epistatic genetic effects, and so on. Breeding programs are always dynamic and required to choose various factors carefully to optimize the genetic gain. Constant improvement and cost reduction of genotyping, as well as genotyping of large breeding populations, may help in the inclusion of distantly related individuals in the TP; GBS could be one such potential genotyping platform. In the last decade, many solutions have been offered to overcome the challenges associated with GS-assisted breeding, but still, there are certain practical difficulties, particularly for complex genome crops (such as sugarcane) and G × E sensitive traits (including yield and component traits). Multi-trait and multi-environment modelling are essential for improving the accuracy to predict the performance of newly developed lines in coming years. Frequently updated prediction models may reduce the prediction accuracy decay, especially when the target population becomes further separated from the TP. Both phenotyping and genotyping have improved progressively over the recent past, and big biological data generation through high-throughput genotyping and phenotyping have contributed to a boom in machine learning and deep learning in commercial agriculture to deliver precision farming strategies.

## Figures and Tables

**Table 1:** Details of genomic selection studies in sugarcane.

Number of Genotypes in Panel	Traits Considered	Number and Type of Marker Used in Genotyping	GS Prediction Models Tested	Prediction Accuracy	Conclusion/Remarks	Reference
167	10 traits: smut, ScYLV, BR, sugar content, bagasse content, bagasse digestibility, plant morphology	1,499 DArT	Bayes LASSO, ridge regression, reproducing kernel Hilbert space (RKHS), partial least square regression (PLSR)	0.13–0.21 (smut), ScYLV, BR, sugar content, 0.16–0.33 (Bagasse content), bagasse digestibility, plant morphology	Equivalent accuracy among the four predictive models for a given trait, and marked differences were observed among traits	[4]
173 F_1_s	Lignocellulosic biomass, disease resistance	21,895 single-dose SNP markers	Additive, dominance, and epistasis model, Bayes A, reproducing kernel Hilbert space (RKHS), support vector machine regression (SVMR)	0.44–0.77	MAS performed reasonably well for traits controlled by a smaller number of QTN The prediction accuracy of MAS dropped dramatically compared with four tested GS models	[47]
Three different panels: 467, 1146, 738	Cane yield, CCS%	Affymetrix Axiom SNP Array 47,803 SNPs	Bayes A, Bayes B, Bayes LASSO, ridge regression	0.25–0.45	Unbalanced dataset, ascertainment bias array	[48]
219	Brown rust	13,458 SNPs	K-nearest neighbor (KNN), support vector machine (SVM), Gaussian process (GP), decision tree (DT), random forest (RF), multilayer perceptron (MLP) neural network, adaptive boosting (AB), Gaussian naive Bayes (GNB)		Increase in predictive power is due to the identification of regions influencing the phenotype, possibly in QTLs or regulatory genomic elements	[49]
3984	Cane yield, CCS%, fibre content, flowering traits	26 K SNP from Affymetrix Axiom 44 K sugarcane SNP chip	GBLUP, genomic single step (GenomicSS), BayesR	Cane yield: 0.3 CCS%: 0.43–0.47 fibre content: 0.43–0.46	The single-step analysis (GenomicSS), is attractive for routine evaluations, as EBV are produced on the same scale for all clones (genotyped or non-genotyped)	[50]
432	Orange rust, brown rust	8825 coding region-based SNPs	Random regression BLUP (RR-BLUP), reproducing kernel Hilbert space (RKHS), random forest (RF), support vector regression (SVR)	0.28–0.43 BR 0.13–0.29 OR	Parametric GS model outperformed non-parametric models	[51]
1000		10,000 SNPs	An additive trait genetic model, ridge regression BLUP (rrBLUP)	0.3–0.5	A combination of improving both additive and non-additive genetic effects holds the potential to improve long-term genetic gain in sugarcane breeding	[52]

**Table 2:** Description and source codes for the important machine and deep learning models for genomic selection in plant breeding programs.

Prediction Model	Description	Code	Reference
Random Forests (RF)	RF uses different sets of nodes, branches, and depth for building a regression-based tree	https://github.com/xuanxu/nimbus (accessed on 31 May 2022)	[139]
Support Vector Machines (SVM)	SVM uses kernel and cost functions to model hyperplane for predictions	https://github.com/afiliot/Kernel-Methods-For-Genomics (accessed on 31 May 2022)	[140]
Partial Least Square (PLSR)	PLSR uses dimensional reduction technique to produce a latent variable, which is ultimately used to make predictions	https://datadryad.org/stash/dataset/doi:10.5061/dryad.7f138 (accessed on 31 May 2022)	[141]
Multilayer Perceptron (MLP)	MLP uses a set of input, hidden, and output layer, with large number of neurons and activation functions to model the trend in the data	https://github.com/saeedkhaki92/Yield-Prediction-DNN (accessed on 31 May 2022)	[127]
Convolutional Neural Network (CNN)	CNN uses a set of convolutional, flattening, pooling, and dense layer for predicting the output	https://github.com/Sandhu-WSU/DL_Wheat (accessed on 31 May 2022)	[127]
DeepGS	DeepGS uses a set of input, convolutional, sampling, and fully connected layer to predict the output	https://github.com/cma2015/DeepGS (accessed on 31 May 2022)	[137]
Recurrent Neural Network (RNN)	RNN mostly uses for predicting longitudinal and time-series-based data	https://figshare.com/s/5cd5e5e4eaeef55b721f?file=24963563 (accessed on 31 May 2022)	[142]
Arc-cosine Kernel (AK)	AK estimates the stepwise covariance matrix in model training	https://www.frontiersin.org/articles/10.3389/fgene.2019.01168/full#h7 (accessed on 31 May 2022)	[143]

## Data Availability

Not applicable.
